# Special issue on “Children's and adolescents' rights to participate in their pain management”

**DOI:** 10.1002/pne2.12116

**Published:** 2023-11-10

**Authors:** Juan Bornman, Stefan Nilsson

**Affiliations:** ^1^ Centre for Augmentative and Alternative Communication University of Pretoria Pretoria South Africa; ^2^ Institute of Health and Care Sciences Sahlgrenska Academy, University of Gothenburg Gothenburg Sweden; ^3^ University of Gothenburg Centre for Person‐Centred Care (GPCC), Sahlgrenska Academy, University of Gothenburg Gothenburg Sweden; ^4^ Queen Silvia Children's Hospital Gothenburg Sweden

**Keywords:** autonomy, children's voices, human rights, pain management, self‐report

## 1 CHILDREN'S OWN VOICES SHOULD BE THE HEARTBEAT OF ANY TYPE OF PAIN MANAGEMENT

The dialogues between children and adolescents and their healthcare providers are essential in managing pain in accordance with the child's interests and wishes. It can, however, be challenging to ensure that pain management is in the child's best interest due to a myriad of reasons. One of these relates to the specific communicative challenges that exist in this population, that might arise because of the child's level of development, including receptive and expressive communication skills, the particular illness and treatment (e.g., tracheotomy), distress, and/or disabilities. It is therefore unsurprising that children and adolescents are regarded as vulnerable groups in pediatric healthcare.

The importance of using person‐centred care has been emphasized in healthcare to facilitate the voices of children and young people and as a way in which their views can be respected. The use of person‐centred care promotes the child and adolescent's narratives and emphasizes the use of shared decision‐making to be the primary source of pain management decisions.^1^ This approach is also in line with the United Nation's Convention on the Rights of the Child,^2^ and specifically with Article 24 that focusses on health. Other articles of this convention also apply. For example, Article 12 highlights respect for children's views and allows them the opportunity to give their opinions freely, specifically about issues that directly affect them. In addition, Article 13 emphasizes children's right to share their thoughts freely in terms of what they learn, think, and feel and that this can be done by talking, drawing, or writing. The nexus between these two articles and person‐centred care is thus clear. Likewise, Article 17, which focusses on access to information from the Internet, radio, television, newspapers, books, etcetera, is equally relevant. Article 17 also urges adults (in this case healthcare providers) to ensure that the information provided to children is not harmful and that it is in a language format that all children can understand.

Previous research has demonstrated that parent's assessments of pain symptoms in children and adolescents differ from the child's and adolescent's own assessment.^3^ Self‐reports of the evaluation of pain management should be the first choice in pediatric healthcare. However, children's rights, as described above, are not always prioritized, and healthcare provider's own measurements often guide the decision‐making around children and adolescents´ pain management.^4^ This means that it is necessary to develop and implement new strategies that optimize shared decision‐making in pediatric healthcare. In an attempt to allow children and adolescents to take on a greater role in their own pain management, new tools and interventions are required to support acute and chronic pain measurement and treatment.

This special issue includes five papers that showcase and expand knowledge about children's rights in pain management. Articles within the special issue contain three different types of methodologies (i.e., a study protocol, a qualitative design, and a scoping review) and include both acute and chronic pain management. In total 49 children and adolescents aged 6–19 years are included in the qualitative studies, giving a broad range of data collection.

The first paper describes a study aimed at providing insight into practical considerations when designing clinical trials, by focussing on two clinical trials for headaches in children.^5^ It specifically focuses on recruitment and retention preferences, potential barriers to research, and optimizing study designs. The participants indicated that they prefer to be contacted directly by their physician about potential studies that they can participate in and that they enjoy developing rapport with study staff. However, time commitment was mentioned as one of the barriers to participation. The findings from this study could contribute to improved study designs for future studies.

The second paper presents a multi‐site project design that combines quantitative and qualitative methods. It describes the design of three different studies and produces a study protocol. The overall aim is to evaluate psychometric properties of the newly developed electronic Faces Thermometer Scale (a scale with 11 grades) for assessing pain in children 8–17 years of age. It concludes by proposing that this new digital pain assessment tool (i.e., the electronic Faces Thermometer Scale) could potentially strengthen the child's voice within pediatric healthcare.^6^

The aim of the study described in paper three is to illuminate restraint from the perspective of children's and adolescent's experiences of feeling forced during medical procedures. The analysis reveals that it hurts to get forced. This is illustrated clearly in the six themes delineated from the results: bodily misery, emotional rebellion, feeling disregarded, feeling physically limited, desiring escape, and leaving deep traces. The findings emphasize that healthcare providers should take action to support children's self‐determination, participation, and integrity in pediatric healthcare.^7^

Paper four illuminate adolescents' experiences of Help Overcoming Pain Early (HOPE), a person‐centred intervention delivered in a school setting by school nurses. The HOPE intervention was built on person‐centred ethics and consisted of four meetings between school nurses and adolescents about stress and pain management. The overarching theme describes how adolescents strived to become themselves again. The study concludes by suggesting that a person‐centred intervention applied in a school context could facilitate confidence in adolescents with chronic pain.^8^

The fifth and final paper focuses on children with autism spectrum disorder and their experience of social interaction and communication challenges. Pain is one of the most complex human stressors, and it remains so when it comes to how children with autism spectrum disorder communicate their pain. This review highlights that children with autism spectrum disorder use different verbal and non‐verbal methods to communicate their pain experiences, calling attention to the importance of using holistic pain assessment strategies to meet the goal of upholding children's rights.^9^

In conclusion, in attempts to foreground child and adolescent perspectives regarding pain measurement and management, at least four aspects should be considered. First, children need to be provided with opportunities to express their opinions, which could include alternative methods of expression, such as drawing or painting. Second, children should be furnished with appropriate means to express themselves for example, using any form of communication, and often using multi‐modal forms of communication. Third, adults (i.e., all healthcare providers) should engage with children in one‐to‐one situations in which their opinions are listened to in a respectful and non‐judgemental way. Fourth, adults should act on the opinions and/or wishes children shared—also related to how they think their pain should best be managed.
